# Why Does Dual-Tasking Hamper Implicit Sequence Learning?

**DOI:** 10.5334/joc.136

**Published:** 2021-01-07

**Authors:** Eva Röttger, Fang Zhao, Robert Gaschler, Hilde Haider

**Affiliations:** 1 Department of Psychology, University of Bremen, Hochschulring 18, 28359 Bremen, DE; 2 Department of Psychology, FernUniversität in Hagen, Universitätsstr. 33, 58084 Hagen, DE; 3 Department of Psychology, University of Cologne, Richard-Strauss-Str. 2, 50931 Köln, DE

**Keywords:** Implicit Sequence Learning, Multitasking, Prediction

## Abstract

Research on the limitations of dual-tasking might profit from using setups with a predictable sequence of stimuli and responses and assessing the acquisition of this sequence. Detrimental effects of dual-tasking on implicit sequence learning in the *serial reaction time task* (SRTT; Nissen & Bullemer, 1987) – when paired with an uncorrelated task – have been attributed to participants’ lack of separating the streams of events in either task. Assuming that co-occurring events are automatically integrated, we reasoned that participants could need to first learn which events co-occur, before they can acquire sequence knowledge. In the training phase, we paired an 8-element visual-manual SRTT with an auditory-vocal task. Afterwards, we tested under single-tasking conditions whether SRTT sequence knowledge had been acquired. By applying different variants of probabilistic SRTT-tone pairings across three experiments, we tested what type of predictive relationship was needed to preserve sequence learning. In Experiment 1, where half of the SRTT-elements were paired to 100% with one specific tone and the other half randomly, only the fixedly paired elements were learned. Yet, no sequence learning was found when each of the eight SRTT-elements was paired with tone identity in a 75%–25% ratio (Experiment 2). Sequence learning was, however, intact when the 75%–25% ratio was applied to the four SRTT target locations instead (Experiment 3). The results suggest that participants (when lacking a separation of the task representations while dual-tasking) can learn a sequence inherent in one of two tasks to the extent that across-task contingencies can be learned first.

Many of our daily activities require fulfilling more than one single task at a moment. In almost all cases, this multitasking inevitably comes along with several sorts of performance costs (for a recent review, see Koch, Poljac, Müller, & Kiesel, 2018). Often, however, at least one of the tasks is sequential in nature (e.g., making coffee or dressing up the children in the morning while talking to our partner, etc.). With some practice, this sequencing might help not to confuse the tasks and to reduce the subjectively experienced effort as well as the objective performance costs. For instance, everyone knows from the first driving lesson how difficult it is to manage all the concurrent and sequential activities needed to drive a car, leaving little capacity to additionally engage in small talk with the driving instructor. With sufficient practice, however, we are capable of talking to our passengers while driving without experiencing any effort. Likely, a large amount of practice with the sequential driving activities helps us to keep “driving” and “talking” separate. In other words, already *knowing* the action sequence within one task quite well might support dual-task performance. An open question is, however, how sequence *learning* (i.e., the acquisition of implicit sequence knowledge) might proceed *while* dual-tasking – especially if there is a tendency to confuse the tasks (cf. Hazeltine & Schumacher, 2016). Indeed, as we will explicate thoroughly below, it has been frequently shown that implicit sequence learning is affected by dual-tasking (for overviews, see Keele, Ivry, Mayr, Hazeltine, & Heuer, 2003; Schumacher & Schwarb, 2009; Schwarb & Schumacher, 2012).

Surprisingly, in the research on dual-tasking, the role of task-inherent regular sequences has drawn only little attention (Schiffer, Waszak, & Yeung, 2015). Rather, in most dual-task paradigms, the events in either task follow a random sequence. These randomly sequenced tasks are well-suited to investigate the potential interference between two concurrently presented tasks within the current trial (e.g., Fischer & Dreisbach, 2015) – and/or as a function of the immediately preceding trial (e.g., Janczyk, 2016). However, with randomly sequenced tasks, it is difficult to discover sources of dual-task costs that come into effect by processing longer – repeating – sequences of trials. These sources may even remain undetected.

For instance, with two simultaneously presented tasks, it might be that costs are caused by parallel response selection (cf. Miller, Ulrich, & Rolke, 2009) – but it is also reasonable to assume that they are caused by a notorious tendency of the human cognitive system to not keep the task representations clearly separate (cf. Hazeltine & Schumacher, 2016). The finding that a regular sequence of events inherent within one task in a dual-task paradigm cannot be learned properly when the other task is random (Röttger, Haider, Zhao, & Gaschler, 2019; Schmidtke & Heuer, 1997) hints at the importance of such task confusion as a source of dual-task costs. In this case, the reduction of sequence learning seems to indicate a lack of separation of the tasks. By implementing a regular sequence of events in only one of the tasks, and by varying the extent of randomness within the other task (i.e., by varying the across-task predictability), the resulting implicit sequence knowledge could possibly serve as a marker of a type of dual-tasking limitations that cannot be assessed when using two randomly sequenced tasks. Such a marker would be conceptually different from (or at least expand) established markers of dual-tasking limitations like, for instance, the *psychological refractory period* (PRP) effect (e.g., Pashler, 1994) or the *backward crosstalk effect* (BCE; e.g., Hommel, 1998; Janczyk, Pfister, Hommel, & Kunde, 2014).

In the present study, our goal was to investigate how dual-tasking – or, more specifically, the predictability of events across the tasks – affects the amount of within-task implicit sequence learning. Therefore, we manipulated the contingencies between the elements of a *serial reaction time task* (SRTT; Nissen & Bullemer, 1987), containing a regular sequence, and the elements of a concurrently presented randomly sequenced tone-discrimination task and compared the resulting amount of implicit SRTT sequence knowledge.

Implicit learning is assumed to be a rather robust learning phenomenon (e.g., Frensch & Stadler, 1998). In the SRTT, a paradigm used to investigate implicit sequence learning, the participants see several marked positions on the screen. Each of these screen locations is spatially mapped to a response key and the participants are asked to press the respective key as soon as the assigned screen location is highlighted. Unbeknownst to the participants, the marked screen locations (and thus the responses) follow a regular sequence. When, after some practice, this sequence is replaced by a random sequence, the response times increase, indicating sequence learning. This sequence learning is thought to be implicit because the participants’ ability to verbally describe the sequence is usually scarce. In addition, implicit sequence learning is thought to occur involuntarily as a consequence of repeatedly responding to the elements belonging to the sequence. More generally, it is assumed to appear simply as a consequence from acting in a rather stable environment and can be interpreted as the adaptation to regularities inherent in the environment without the need for conscious awareness about the ongoing learning process itself or the learned content (Cleeremans, 2011).

Despite its robustness, implicit sequence learning has been shown to be disrupted under some – but not all – dual-task conditions (see Keele et al., 2003; Schumacher & Schwarb, 2009; Schwarb & Schumacher, 2012). In the field of implicit learning, such decrements have often been attributed to limited attentional resources that have to be shared between the two tasks (e.g., Curran & Keele, 1993; Jiménez & Méndez, 1999; Nissen & Bullemer, 1987). Yet, it has already been proposed by Keele et al. (2003) that, alternatively, the learning decrements in dual-tasking might result from the random secondary task disturbing the coherence of the to-be-learned sequence (see also Rah, Reber, & Hsiao, 2000; Schmidtke & Heuer, 1997; Stadler, 1995).

Some researchers have started to use implicit learning paradigms also in the field of dual-tasking (Ewolds, Broeker, de Oliveira, Raab, & Künzell, 2017; Gaschler et al., 2018; Gaschler, Zhao, Röttger, Panzer, & Haider, 2019; Halvorson, Wagschal, & Hazeltine, 2013; Röttger et al., 2019; Schumacher & Schwarb, 2009; Zhao, Gaschler, Nöhring, Röttger, & Haider, 2020; Zhao et al., 2019). The focus within this research field is slightly different from that in the field of implicit learning. Rather than asking whether implicit learning depends on attentional resources, the primary question here concerns the origin of dual-task costs. As mentioned above, one important advantage of combining implicit sequence learning with dual-tasking paradigms might be that it allows to investigate the costly interplay of processing the within- and across-task contingencies while dual-tasking.

A re-interpretation of the results of a study by Schumacher and Schwarb (2009) might serve as an example. The authors investigated the effects of a (random) concurrent tone-discrimination task on implicit sequence learning in a SRTT by manipulating the time interval between the two stimuli (*stimulus onset asynchrony*; SOA) in a between-subjects design. In one condition, they presented both stimuli, the visual SRTT target and the tone, simultaneously (SOA = 0 ms), while in the other condition, the tones appeared consistently 750 ms after the SRTT target (SOA = 750 ms). In both conditions, the participants’ task was to manually respond to the SRTT target and to verbally respond to the tone. After several blocks of training, it turned out that implicit sequence learning in the SRTT was reduced in the condition with simultaneous stimulus presentation, but preserved in the condition with the long SOA. In a second experiment, the participants were instructed to either prioritize the SRTT or to give both tasks equal priority, while in both conditions the stimuli were presented simultaneously. The results showed preserved implicit sequence learning only in the SRTT priority condition. From these findings, the authors concluded that it is the simultaneous response selection process, which impedes implicit sequence learning in dual-task contexts (without further specifying the exact mechanism). This conclusion is in line with the more general assumption held in the field of dual-tasking that (some) dual-task costs result from parallel response selection in both tasks (e.g., Lehle & Hübner, 2009; Logan & Gordon, 2001). However, given the concurrency of stimulus presentation (i.e., with SOA = 0 ms), an alternative interpretation of the results of Schumacher and Schwarb (2009) might be that, when the participants were instructed to give the two tasks equal priority, they integrated the two tasks into one single task set or “task file” (Hazeltine, Ruthruff, & Remington, 2006; Hazeltine & Schumacher, 2016; Schumacher et al., 2018) thereby confounding the regular SRTT sequence with random elements.

This assumption is supported by results of Halvorson, Wagschal, et al. (2013). The authors presented two groups of participants with the same dual-task (i.e., one visual-manual SRTT and a concurrent randomly sequenced two-choice auditory-manual task) and solely manipulated the participants’ representations of the task context by instruction.^1^ If the participants viewed the two tasks as one single integrated task, implicit sequence learning in the SRTT was disturbed. By contrast, representing the two tasks as separate preserved implicit sequence learning (see also, e.g., D’Angelo, Milliken, Jiménez, & Lupiáñez, 2014).

The findings of Freedberg, Wagschal, and Hazeltine (2014) hint at a potential mechanism responsible for the impairment of *within-task* sequence learning due to task integration in dual-task contexts by demonstrating that whenever the participants viewed two simultaneously presented tasks as belonging together, the contingencies *between the tasks* were learned (even without overlapping task features). If this focus on across-task events turned out to be the default mode of associative learning in dual-task contexts, it is conceivable that a sequence within a task can only be learned if contingencies between the tasks can be learned in advance.^2^ Thus, alternatively to the assumption of parallel response selection put forward by Schumacher and Schwarb (2009), these studies suggest that a general problem in dual-tasking might be that the participants sometimes generate one single integrated task set, rather than two separate task sets – which is harmful for sequence learning if the additional task is random.^3^

First evidence that task integration might be a crucial factor for the impairment (as well as the preservation) of implicit sequence learning in dual-task situations was already reported in a study of Schmidtke and Heuer (1997). They paired a (visual-manual) SRTT containing a regular 6-element sequence with an auditory-motor go/no-go task, containing either also a 6-element, or a 5-element, or a random sequence (D-6, D-5, and D-R condition; “D” refers to “dual-task”). They suggested a mechanism that integrates the visual and the auditory stimuli into one sequence resulting in either a 12-element, a 60-element or an indefinitely long sequence in the three conditions.^4^ They assumed that the resulting implicit sequence knowledge should be the more disturbed the longer the integrated sequence (which implies that the two sequences are also less correlated).

Sequence knowledge assessed in dual- as well as single-task tests, supported their assumption of task integration (Schmidtke & Heuer, 1997; Experiment 1). In the single-task test, in which only the SRTT was presented and the RTs in one random block were compared to the RTs in two surrounding sequence blocks, knowledge of the pure SRTT sequence was moderately present in all three conditions (i.e., longer RTs in the random- than in the sequence blocks).^5^ However, in the dual-task test (with the tone-task still present), the size of the implicit learning effect was very different in the three conditions: The implicit learning effect was very small in the D-R condition (and smaller than in the single-task test). It was intermediate (and as large as in the single-task test) in the D-5 condition, and, most importantly, very large (and larger than in the single-task test) in the D-6 condition. Thus, the disruption of implicit sequence learning was indeed higher the longer the integrated sequence. This outcome also fits in with the assumption that the participants did not represent the boundaries between the two tasks in a dual-task paradigm as sharply delimited.

Recently, Röttger et al. (2019) added some more evidence for the assumption of task integration as a crucial factor causing costs in dual-tasking situations. They combined a visual-manual SRTT with an auditory-vocal tone-discrimination task. Differently from the go/no-go procedure used by Schmidtke and Heuer (1997), the tone-task required a vocal response for each of the two tones (cf. Schumacher & Schwarb, 2009). Further, they assessed the acquired implicit sequence knowledge exclusively within SRTT single-task tests. In the correlated-tasks condition (Experiment 2), the 8-elements 2^nd^ order SRTT sequence (3-1-2-4-1-3-4-2) was combined with a regular tone sequence that was twice as long (16 elements). Thus, the integrated sequence of manual and vocal responses had 32 elements – lying in between the D-6 (12 elements) and the D-5 (60 elements) sequence of Schmidtke and Heuer (1997).^6^ Despite the slight methodological differences, Röttger et al. could replicate the original findings. The single-task test revealed that the participants had learned the SRTT-sequence.^7^ By contrast, when the identical SRTT sequence was paired with a random tone-task (see Experiment 1), the single-task test revealed strongly reduced sequence learning.

One further experiment of the Röttger et al. (2019) study is noteworthy in this context. In Experiment 4, four of the eight SRTT-elements had been fixedly paired with either the high or the low-pitched tone while the other four elements had been randomly paired with the two tones. The single-task implicit learning test conducted after the dual-task training blocks revealed that exclusively these (formerly) fixedly paired sequence positions had been learned. In addition, this finding was replicated using an offline knowledge test (i.e., the post-decision wagering task; Haider, Eichler, & Lange, 2011). This test allows assessing whether participants indeed possess knowledge about which SRTT-element occurs at each particular sequence position. The results showed that this was the case for all four (formerly) fixedly paired SRTT-positions, but for none of the (formerly) variably paired positions. At first glance, this finding sounds a bit counterintuitive because it suggests that the participants did not learn the associations between the SRTT-elements (chaining). Rather, for the fixedly-paired elements only, they had acquired associations between the ordinal sequence positions and the respective SRTT-elements (e.g., at the first position, the SRTT-element 3 appears, etc.). In the sequence learning literature, this type of learning is termed ordinal position learning (see, e.g., Lashley, 1951; Schuck, Gaschler, & Frensch, 2012; Schuck, Gaschler, Kreisler, & Frensch, 2012; Terrace, 2005). In the single-task test, faster responses were observed when the formerly fixedly paired SRTT-elements occurred at their correct ordinal positions. When the formerly variably paired SRTT-elements occurred at the correct position in the sequence, no such benefit was observed. This is surprising as these SRTT-elements could have been unequivocally predicted by the preceding fixed SRTT-tone pairing.

Taken together, the reported findings on implicit sequence learning under dual-tasking conditions suggest that the participants in dual-tasking tend to conceptualize the two tasks as belonging together and to integrate them into one single task set – at least when the tasks are presented simultaneously and without further instructions to keep the representations separate (for similar assumptions, see Freedberg et al., 2014; Halvorson, Ebner, & Hazeltine, 2013; Hazeltine & Schumacher, 2016; Schumacher et al., 2018). This common representation might then lead to the generation of associations across the two tasks. If the co-occurring elements are contingently paired, participants can acquire SRTT-tone compounds. In a next step, these can become associated across the trials resulting in implicit sequence learning. If, however, the SRTT-elements and the tones are randomly paired, no compounds can be learned. This in turn hampers sequence learning in setups in which participants fail to clearly separate the tasks and process them in an integrated manner instead.

This proposal differs from the assumption of Schmidtke and Heuer (1997). They seem to suggest that the associations between the SRTT-elements and the tones (within a trial) are learned simultaneously with the associations between the tones and the SRTT-elements (across trials). According to this view, the resulting length of the integrated sequence should determine whether sequence learning will occur. By contrast, if the within-trial SRTT-tone pairings have to be learned at first, the length of the integrated sequence might be of minor importance for implicit sequence learning under dual-tasking conditions. Instead, it should matter how easy it is to learn these temporally close co-occurring events. Only after having learned these across-task contingencies, the formation of associations between the temporally separate successive events within the SRTT should be possible. The three experiments reported below aimed at investigating this hypothesis.

## THE PRESENT STUDY

The present study builds on Experiment 4 of the Röttger et al. (2019) study mentioned above. The observations in this experiment suggested that the participants tend to represent the two tasks within one single task set leading to a potentially costly interplay of acquiring within- and across-task associations while dual-tasking. Therefore, we started with a replication of this experiment, realizing slight methodological modifications. The repeating SRTT sequence in the original Experiment 4 had a length of eight elements (or sequence positions). As there were four different target locations on the screen (numbered from 1 to 4 from left to right), the target occurred twice at each location within one sequence loop (with a different predecessor and successor, respectively). Such a sequence is characterized as a 2^nd^ order conditional sequence. That is, in order to be able to fully predict an upcoming SRTT-element, it needs to take two preceding elements into account.

In the original experiment, one of the four target locations of the 2^nd^ order SRTT sequence (3-1-2-4-1-3-4-2) had been inadvertently fixedly paired with one specific tone at both sequence positions (target location 1). Another target location (target location 2) had been always randomly paired with the two tones at both sequence positions. Target location 1 might thus have served as a salient starting point for the sequence (as an anchor) and as such might have facilitated ordinal position learning (e.g., Schuck, Gaschler, Kreisler, et al., 2012) instead of associative chaining (i.e., learning the transitions within the SRTT). In order to exclude this alternative explanation for the finding of ordinal position learning, we combined here each of the four target locations of the original SRTT sequence once fixedly and once randomly with the tones. Consequently, none of the eight sequence positions should have been privileged to serve as an anchor. A replication of our former finding that ordinal positions were learned but associative chaining among the SRTT-elements was absent would, thus, hint at the primacy of learning the across-task contingencies in dual-task implicit sequence learning contexts. A replication would also lay the foundation for Experiments 2 and 3.

The goal of Experiments 2 and 3 was to further investigate how exactly the presence of across-task contingencies affects implicit sequence learning in dual-tasking. In both experiments, we used the 8-elements 2^nd^ order sequence of Experiment 1 and combined each sequence element with a probability of 75% with a certain tone. The only difference between the two experiments was that in Experiment 2, we realized the contingencies between the eight sequence positions and the respective tones leading to the following combined SRTT-tone sequence: 3H-1L-2L-4L-1H-3L-4H-2H (H refers to high, L to low pitched tones; the numbers 1 to 4 represent the different target locations). By contrast, in Experiment 3, we realized the contingencies between the four different target locations (1–4) and the respective tones (3H-1H-2L-4L-1H-3H-4L-2L). Thus, the crucial difference between the two sequences was that in the former, each SRTT target location occurred equally frequently with either the high or the low tone, depending on the respective sequence position, whereas in the latter, one SRTT target location occurred frequently with the same tone at both sequence positions. Importantly, however, the length of the integrated sequences in both experiments was identical (8 × 2 = 16 elements) because each sequence element was contingently paired with a certain tone. According to the assumption of Schmidtke and Heuer (1997), the sequences in both experiments should be learned to a similar extent. However, if the across-task contingencies between the SRTT-elements and the tones have to be learned in advance, before any learning of the within-task contingencies (i.e. the associations between the SRTT-elements) can occur, it should be harder to learn the SRTT sequence in Experiment 2. Here, the participants have to learn eight different SRTT-tone compounds and, in addition, at which ordinal sequence position a specific target location appears together with the high tone and at which position together with the low tone. By contrast, in Experiment 3, they had to learn only four different SRTT-tone compounds.

Following the approach of Rah et al. (2000), we focused primarily on the question whether these manipulations of the across-task contingencies differentially disturb implicit sequence learning. Therefore, we report them as separate experiments to avoid the occurrence of non-interpretable interactions. Furthermore, given this goal of the study, our focus lies primarily on the SRTT data (RTs and error rates). The tone-task should be seen mainly as a part of the contingency manipulation. However, since it is highly likely that these manipulations also affect the performance in the tone-task, we will also report the data from this task (RTs only).

## EXPERIMENT 1

Experiment 1 aimed at replicating Experiment 4 of the Röttger et al. (2019) study. For this purpose, we combined each of the four different target locations of the SRTT once fixedly and once randomly with the tones, depending on its position within the sequence. Replicating our former finding of ordinal position learning of only the fixedly paired SRTT-elements would strengthen our conjecture that implicit sequence learning in a dual-tasking situation presupposes that the across-task contingencies have to be learned first.

## METHOD

### PARTICIPANTS

Twenty-five students (8 men) of the University of Cologne (mean age 23.08, *SD* = 3.55) participated in the experiment either for monetary compensation or for course credit. Each session lasted approximately 45 min.

### APPARATUS AND STIMULI

The experiment was controlled by custom-written software (Lazarus/FreePascal, compiled for Microsoft Windows). Placeholders for the visual SRTT target (an uppercase “X”) were four horizontally aligned white squares on a light grey background (100 × 100 pixels, separated by gaps of also 100 pixels). They were displayed slightly below the center of a TFT monitor (19 inch; 1280 × 1024 pixels) that was connected with a standard PC. In each trial, the SRTT target occurred for 100 ms in one of the four white squares and the participants had to press the respective spatially mapped key in response (Y, X, N, M on a German QWERTZ-keyboard). Unbeknownst to the participants, the response locations of the SRTT followed a 2^nd^ order conditional 8-elements sequence (3-1-2-4-1-3-4-2). In the dual-task trials, a high (900 Hz) or a low (300 Hz) pitched tone, lasting 56 ms, was played simultaneously, requiring the respective verbal responses “hoch” or “tief” [high vs. low]. Both tones occurred equally frequently during the dual-task training blocks. Crucially, across one SRTT loop, each of the four target locations was once fixedly paired with a particular tone and once randomly paired with the tones (3F-1F-2R-4F-1R-3R-4R-2F; F = fixedly paired, R = randomly paired).^8^

A sound mixer (Behringer XENYX 302USB) served as a bridge between headset and PC and integrated the tone stimuli with the verbal responses into one single wave-file per trial. The tone-task was analyzed offline, after the experiment.

### PROCEDURE

All participants were introduced step by step into the dual-task training phase. After 20 practice trials with only the tone-discrimination task and another 20 practice trials with only the SRTT, they received 20 practice trials with the dual-task setup. In this first phase, both tasks did not follow any regular sequence.

In the training phase, the participants performed 6 dual-task blocks of 96 trials each. Now, the SRTT followed the 8-element sequence, each block starting at a randomly drawn sequence position. A dual-task trial began with the presentation of the visual SRTT target (the “X”) at one of the four different target locations and, simultaneously, one of the two different auditory stimuli of the tone-discrimination task. The instructions highlighted equal priority of both tasks and free response order. The response-window closed 2000 ms after the SRTT target onset and the next trial started immediately.

The dual-task training phase was followed by 3 single-task test blocks of also 96 trials presenting only the SRTT. In blocks 7 and 9, the SRTT sequence was replaced by a (pseudo-) randomized sequence (i.e., immediate repetitions were not allowed). In block 8, the originally trained sequence was reintroduced. To allow the participants a short phase of accommodation to the single-task context (and controlling for initial speed-accuracy trade-offs), only the second half of block 7 entered the analysis of the single-task test.

At the end of the experiment, the participant’s explicit sequence knowledge was assessed (for details, see Röttger et al., 2019). Since it turned out that infrequent signs of partly explicit knowledge did not modulate any effect, the respective results will not be reported.

## RESULTS AND DISCUSSION

**Figure A1:**
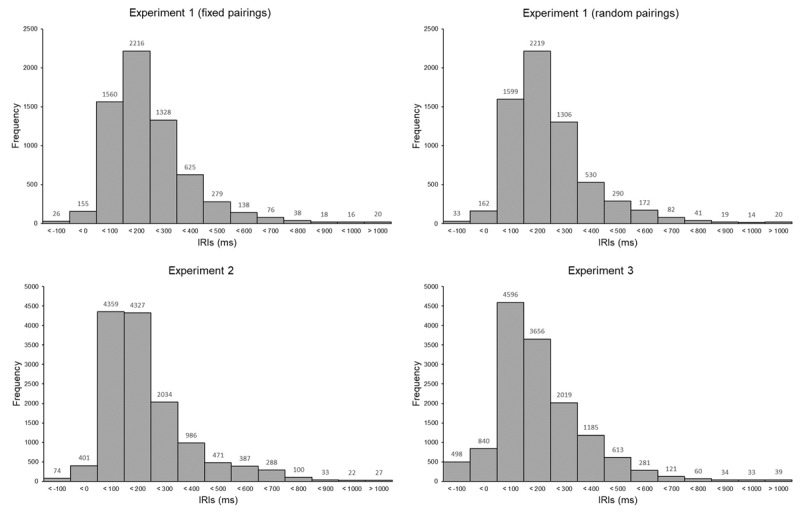
Distribution of the inter-response intervals (IRIs) in the dual-task training phase of the 3 Experiments. In the first bin (i.e., <–100), the minimum IRI is –1655 ms (Experiment 1). In the last bin (i.e., >1000) the maximum IRI is 1811 ms (Experiment 3).

Trials were excluded due to SRTT errors (1.6%) or when the RTs were shorter than 200 ms or longer than 1500 ms in the SRTT (0.5%). As some trials fulfilled multiple exclusion criteria, overall 1.7% of the trials were excluded. We will first report the results of the dual-task training phase and, second, the results of the single-task test phase. The mean inter-response intervals (IRIs), computed as RT_tone-task_ – RT_SRTT_, were positive (197 ms with the fixedly paired SRTT positions/196 ms with the randomly paired SRTT positions) indicating that the participants had predominantly responded to the SRTT first (see also ***Figure A1*** in the Appendix).

### PERFORMANCE IN THE TRAINING BLOCKS

***Table 1*** displays the mean RTs in the SRTT and in the tone-discrimination task for fixedly vs. randomly paired SRTT-elements as a function of block. The mean RTs of both tasks suggest that the participants became generally faster across the six training blocks. In addition, they responded faster (in both tasks) when the sequence position of the SRTT was fixedly paired with the tone than when it was randomly paired.

**Table 1:** Mean RTs and SDs in the SRTT and the tone-discrimination task as a function of block and type of SRTT-element (fixedly vs. randomly paired with the tones) in Experiment 1.


TYPE OF SRTT-ELEMENT	SRTT				TONE-TASK			
	
	FIXED		RANDOM		FIXED		RANDOM	
			
	MEAN	SD	MEAN	SD	MEAN	SD	MEAN	SD

Block 1	554	108	560	106	744	114	754	120

Block 2	524	82	542	88	719	129	731	132

Block 3	506	73	515	70	685	104	710	122

Block 4	489	74	507	70	692	127	708	124

Block 5	477	77	494	74	679	133	690	127

Block 6	461	82	477	74	670	132	684	134

Regular Block 8	410	70	427	54				

Random Blocks 7/9	431	50	431	50				

Learning Effect	21	35	4	23				


The two 6 (block) × 2 (type of SRTT position: fixedly vs. randomly paired) repeated measures ANOVAs (one for each task) with RTs as dependent variable confirmed this by significant main effects of block in the SRTT, *F*(5,120) = 17.03, *p* < .001, \eta _p^2 = .415, and in the tone-task, *F*(5,120) = 9.31, *p* < .001, \eta _p^2 = .280. Also, the effect of type of SRTT position was significant in the SRTT, *F*(1,24) = 18.27, *p* < .001, \eta _p^2 = .432, and in the tone-task, *F*(1,24) = 20.58, *p* < .001, \eta _p^2 = .462. In the SRTT, the participants responded 14 ms faster to the fixedly- than to the randomly paired SRTT-elements. In the tone-task, this difference was 15 ms. The interaction between block and type of SRTT position was not significant (*F*s < 1.56, *p*s > .17).

The SRTT error rates were lower for the fixedly paired SRTT positions (0.90%) than for the randomly paired SRTT positions (2.19%). The corresponding 6 (block) × 2 (type of SRTT position) repeated measures ANOVA with SRTT error rates as dependent variable revealed a significant effect of type of SRTT position, *F*(1,24) = 20.58, *p* < .001, \eta _p^2 = .462 (all other *F*s < 1).

### PERFORMANCE IN THE TEST BLOCKS

**Figure 1:**
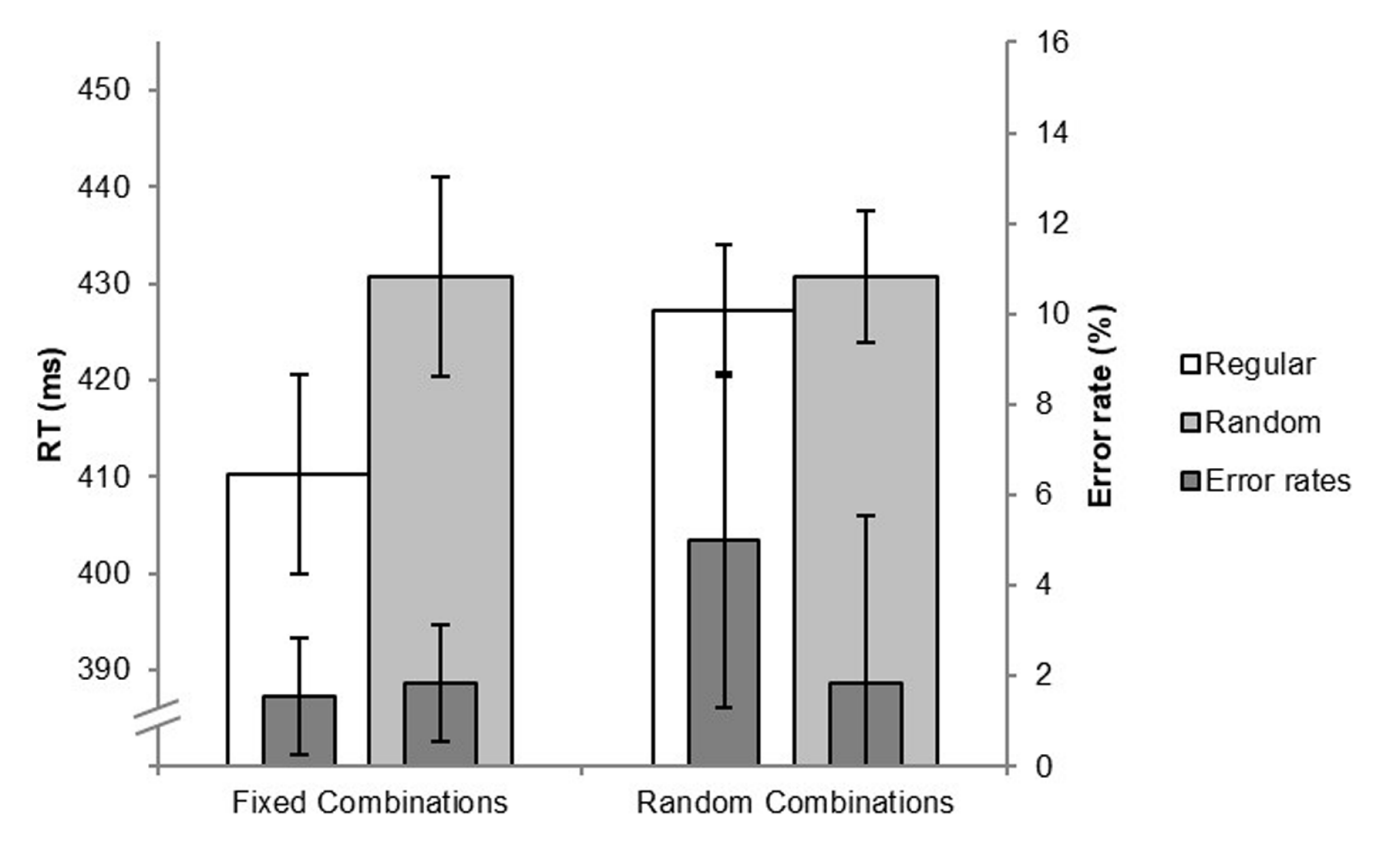
Mean RTs (left y-axis) and error rates (right y-axis) in the regular and the random single-task SRTT test blocks shown separately for SRTT-elements that had been fixedly vs. randomly paired with the tones during the training phase of Experiment 1. Error bars represent the 95% within-subjects confidence intervals of the learning effects (Loftus & Masson, 1994).

To assess implicit learning in the SRTT single-task test, we compared the mean RTs (and error rates) of the collapsed random blocks 7 (2^nd^ half) and 9 with those of the regular block 8. ***Figure 1*** depicts the results of the SRTT single-task test separately for the formerly fixedly- and the formerly randomly paired SRTT positions. For the fixedly paired elements, the mean RT of the random blocks was 21 ms longer than the mean RT of the regular block 8. By contrast, for the randomly paired elements, this difference was only 4 ms. The two respective (two-tailed) *t*-tests revealed that the learning effect for the fixedly paired SRTT positions was significant, *t*(24) = 2.91, *p* = .008, *d* = 0.582, while for the randomly paired elements it was not (|*t|* < 1).

In addition, we conducted Bayes tests (see Dienes, 2014) to assess whether these learning effects indicate evidence for the Null hypothesis or for the alternative hypothesis (no learning vs. learning). Therefore, we specified from our former experiments the learning effect of 26 ms in the single-task condition (Röttger et al., 2019; Experiment 1) as the maximum potential learning effect. For the formerly fixedly paired SRTT positions, the Bayes factor was *BF* = 26.90 indicating clear evidence for the alternative hypothesis. For the formerly randomly paired elements, the Bayes factor was *BF* = 0.36. In a strict sense, this factor indicated insensitivity of the data. Since it was close to the criterion of 0.33 (see, e.g., Dienes, 2014), we are inclined to suspect that indeed no implicit learning had occurred for the randomly paired SRTT-elements.

***Figure 1*** also shows that the error rates were only slightly different in the regular vs. the collapsed random test blocks for the formerly fixedly paired SRTT-elements (1.56% vs. 1.83%, respectively). The corresponding (two-tailed) *t*-test did not reach the level of significance (*t* < 1). For the formerly randomly paired SRTT-elements, the error rates were higher in the regular- (5%) than in the random blocks (1.83%), but this difference was also not significant (|*t*| = 1.25).

Taken together, the findings show that despite none of the SRTT-tone pairings had been especially salient in the current experiment, we could replicate the former findings of Röttger et al. (2019). In the single-task test, we found substantial implicit learning effects only for those SRTT-elements that had been formerly fixedly paired – but not for the formerly randomly paired SRTT-elements. This suggests that the participants had learned associations between the ordinal positions and the fixedly paired SRTT-elements (or SRTT-tone combinations). That means, the participants acquired the knowledge, that, for instance, the target location 1 together with the high tone occurred at the second sequence position. However, they did not learn the transitions between the fixed SRTT-tone combinations and the respective subsequent SRTT-elements. If this had been the case, we should have found substantial learning effects also for directly following randomly paired SRTT-elements. During training, we found shorter RTs in both tasks for the fixed- than for the random SRTT-tone pairings. Interestingly, this was already the case in the very first block suggesting that the across-task contingencies were learned very fast (see also Zhao et al., 2020). We exploratorily followed up on this finding (see the Appendix).

On the basis of these results, we will now turn to our main question how such across-task contingency learning affects within-task implicit sequence learning in a dual-tasking situation. For this purpose, we manipulated the contingencies between the tones and either the SRTT sequence positions (1–8; Experiment 2) or the SRTT target locations (1–4; Experiment 3). In both experiments, the contingency between each single SRTT-element and the respective tone was set to 75% (instead of 100% vs. 50% as in Experiment 1). This means that the participants in both experiments could learn an integrated SRTT-tone sequence of length 16 (eight sequence positions, each combined with one of two tones), irrespectively of whether the SRTT-tone contingencies depended on the eight sequence positions or on the four target locations. Thus, if the length of the integrated sequence is the key factor determining whether implicit sequence learning in dual-tasking situations can take place (Schmidtke & Heuer, 1997), the amount of implicit learning should not differ between the two experiments.

The prediction is, however, quite different when taking across-task contingency learning into consideration. If the participants have to learn at first the contingencies between the SRTT-elements and the tones, it should matter whether the identical SRTT-element (i.e., target location) is bound to one tone at one sequence-position and to the other tone at the other sequence position (3H-1L-2L-4L-1H-3L-4H-2H; H refers to high, L to low pitched tones), or whether the SRTT-tone combination is identical at both sequence positions (3H-1H-2L-4L-1H-3H-4L-2L). In the former case, the participants not only have to learn the SRTT-tone pairings, but also when to expect the same SRTT-element together with the low- and when with the high tone. In contrast, in the latter case this extra learning is unnecessary. Thus, according to the across-task contingency learning hypothesis, no or highly reduced implicit learning should be found when realizing the contingencies between the SRTT sequence positions and the tones (Experiment 2). In contrast, implicit learning should be preserved when the contingencies were realized between the SRTT target locations and the tones (Experiment 3).

## EXPERIMENT 2

Experiment 2 aimed at investigating whether implicit learning is preserved when the contingencies between the elements of the SRTT and the tone-task are realized on the basis of the eight positions of the 2^nd^ order SRTT sequence. In this case, the resulting integrated sequence comprises eight different SRTT-tone pairs. If the participants learn the SRTT-tone contingencies concurrently within- and across the trials, this manipulation should preserve sequence learning. If, however, our assumption is correct that the participants first have to learn the contingencies between the SRTT-elements and the tones within the same trial, we should find strongly reduced sequence learning.

## METHOD

### PARTICIPANTS

Twenty-five students (5 men) of the University of Cologne (mean age 22.72, *SD* = 3.41) participated in the experiment either for monetary compensation or for course credit. Each session lasted approximately 45 min.

### APPARATUS AND STIMULI

Apparatus, stimuli and the 2^nd^ order SRTT sequence (3-1-2-4-1-3-4-2) were the same as in Experiment 1. The only difference concerned the manipulation of the across-task contingencies as described below.

### PROCEDURE

The overall procedure was the same as in Experiment 1. Again, both tones occurred overall equally frequently within the dual-task training blocks. The main difference was that each of the eight SRTT sequence positions (1–8) now appeared together with one particular tone with a probability of 75%. Thereby, depending on its sequence position, each SRTT target location (1–4) occurred frequently together with either the high tone or the low tone (frequent SRTT-tone combinations, hereafter; 3H-1L-2L-4L-1H-3L-4H-2H). In 25% of the trials, the respective infrequent tone occurred (infrequent SRTT-tone combinations, hereafter). Since these frequent and infrequent SRTT-tone pairings occurred randomly during training, it was not possible to assess learning effects separately for both types of pairings in the single-task test (as it was possible in Experiment 1). Therefore, we assessed the extent of implicit learning across the whole SRTT sequence in Experiments 2 and 3.

## RESULTS AND DISCUSSION

As in Experiment 1, trials were excluded due to SRTT errors (1.8%) or RTs < 200 ms or > 1500 ms in the SRTT (0.4%). As some trials fulfilled multiple exclusion criteria, overall 1.9% of the trials were excluded. We will first report the results of the dual-task training phase and, second, the results of the single-task test phase. The mean IRIs were, again, positive (181 ms) indicating that the participants had predominantly responded to the SRTT first (see also ***Figure A1*** in the Appendix).

### PERFORMANCE IN THE TRAINING BLOCKS

***Table 2*** shows the mean RTs in the SRTT and in the tone-discrimination task for the frequent (75% probability) vs. infrequent (25% probability) SRTT-tone combinations as a function of block. As can be seen, the participants became generally faster across the six training blocks in both tasks. In addition, they were also faster (in both tasks) with the frequent than with the infrequent occurring tones.

**Table 2:** Mean RTs and SDs in the SRTT and the tone-discrimination task for the frequent (75% probability) vs. the infrequent (25% probability) SRTT-tone combinations as a function of block in Experiment 2.


SRTT-TONE COMBINATIONS	SRTT				TONE-TASK			
	
	FREQUENT (75%)		INFREQUENT (25%)		FREQUENT (75%)		INFREQUENT (25%)	
			
	MEAN	SD	MEAN	SD	MEAN	SD	MEAN	SD

Block 1	552	104	568	111	717	111	746	107

Block 2	540	96	551	104	719	100	742	103

Block 3	521	99	534	108	702	103	726	100

Block 4	499	81	517	91	678	109	693	108

Block 5	496	87	515	90	674	102	697	105

Block 6	480	73	488	81	663	107	688	113

Regular Block 8	410	53						

Random Blocks 7/9	415	39						

Learning Effect	4	20						


As in Experiment 1, we conducted two separate 6 (block) × 2 (tone frequency: frequent vs. infrequent) repeated measures ANOVAs with mean RTs as dependent variable, one for the SRTT and one for the tone-task. These ANOVAs revealed a significant main effect of block in the SRTT, *F*(5,120) = 19.57, *p* < .001, \eta _p^2 = .449, and in the tone-task as well, *F*(5,120) = 10.94, *p* < .001, \eta _p^2 = .313. Additionally, the RTs were significantly shorter with the frequent (predictable) tones in both the SRTT (14 ms), *F*(1,24) = 24.50, *p* < .001, \eta _p^2 = .505, and also in the tone-task (23 ms), *F*(1,24) = 41.95, *p* < .001, \eta _p^2 = .636. The effect of tone frequency was additive to the effect of block (*F*s < 1 for the respective two-way interactions).

The SRTT error rates were rather low (1.44% and 1.33%) and did not differ across the blocks or with the frequent vs. infrequent tones (all *F*s < 1.18).

### PERFORMANCE IN THE TEST BLOCKS

**Figure 2:**
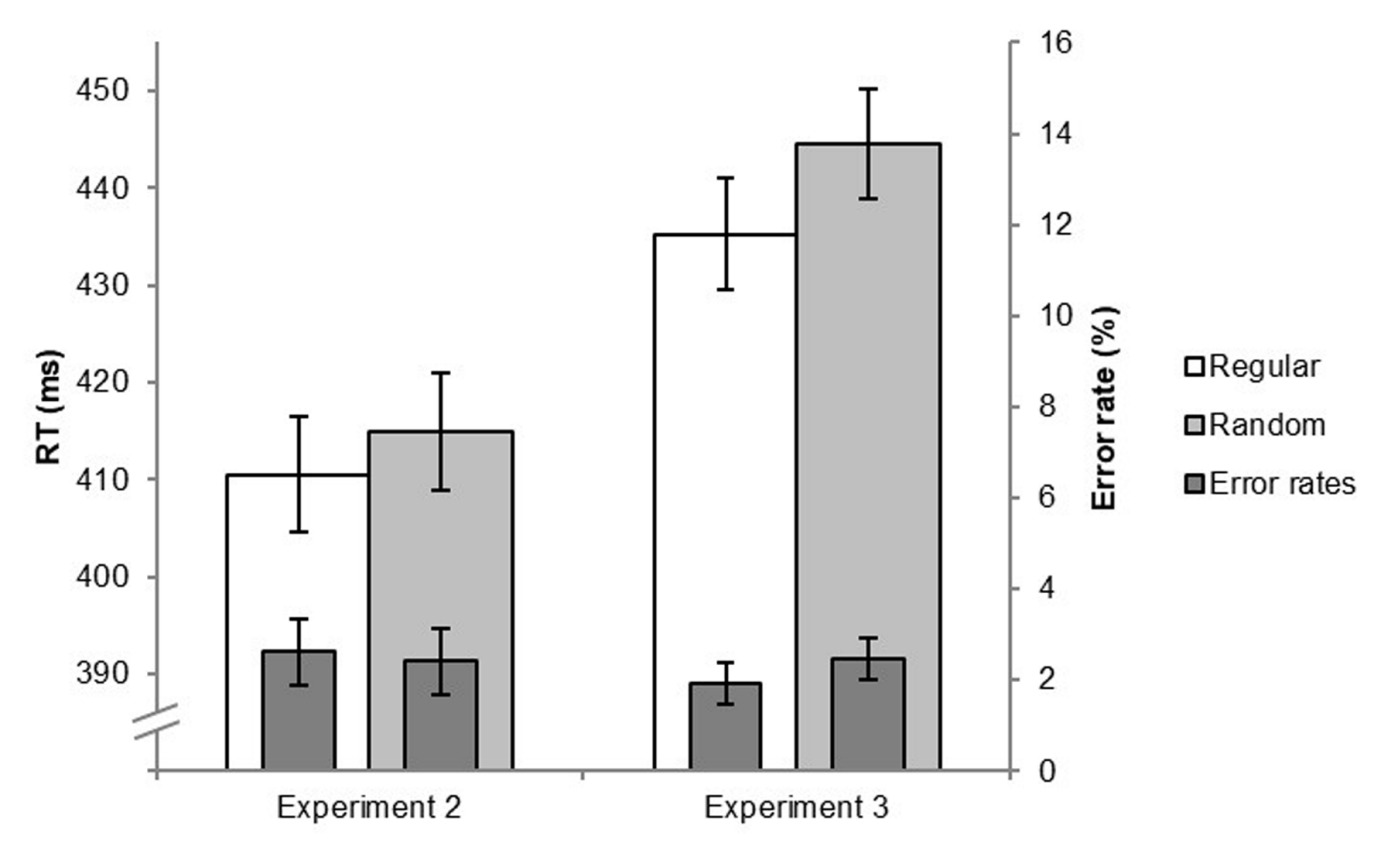
Mean RTs (left y-axis) and error rates (right y-axis) in the regular and the random single-task SRTT test blocks in Experiments 2 and 3. Error bars represent the 95% within-subjects confidence intervals of the learning effects in each experiment (Loftus & Masson, 1994).

***Figure 2*** presents the mean RTs (and error rates) in the collapsed random blocks 7 (2^nd^ half) and 9 and in the regular block 8 in the single-task test phase. The participants responded only 4 ms faster in the regular than in the random blocks suggesting that the SRTT sequence had not been learned. Accordingly, the respective (two-tailed) *t*-test failed to reach the level of significance, *t*(24) = 1.08, *p* = .289, *d* = 0.217.

In addition, we conducted again a Bayes test (see Dienes, 2014) to assess whether this small and non-significant effect indicates evidence for the Null hypothesis (no sequence learning). Based on the effect of 26 ms for the single-task condition in our previous study (Röttger et al., 2019; Experiment 1), as specifying the maximum expected learning effect, the Bayes factor was *BF* = 0.48 indicating insensitivity of the data for making a clear decision.

***Figure 2*** also shows that the error rates only slightly differed between the regular and the collapsed random test blocks (2.63% vs. 2.42%, respectively). The corresponding *t*-test (two-tailed) was not significant (|*t*| < 1).

Overall, Experiment 2 yielded two findings: (1) During training, the participants responded faster in both tasks when the frequent tones were combined with the respective SRTT-elements. This suggests that they developed, again very early, an expectation for the frequent sequence position-tone combinations. (2) Nevertheless, in the single-task test phase we, at best, found a strongly reduced implicit sequence learning effect.^9^ As argued above, such a finding is to be expected if learning the transitions between the SRTT-elements presupposes that the eight SRTT-tone compounds have to be learned first. If, on the other hand, associative chaining of the SRTT-elements occurred concurrently with the learning of the across-task contingencies, the learning effect should have been larger.

However, an alternative possibility is, of course, that participants cannot at all implicitly learn a sequence when the probability of the contingencies between the SRTT-elements and the tones is only 75%. Experiment 3 will shed some light on this alternative explanation.

## EXPERIMENT 3

In Experiment 3, the participants were trained with the same combination of an 8-element 2^nd^ order SRTT and the two-choice tone-discrimination task. In contrast to Experiment 2, now each SRTT target location (1–4) was combined with a particular tone (3H-1H-2L-4L-1H-3H-4L-2L) with a probability of 75%. Again, in 25% of the trials these frequent combinations were changed. If learning the across-task contingencies is necessary for sequence learning to occur in dual-tasking situations, our single-task test should reveal a significant learning effect.

## METHOD

### PARTICIPANTS

Twenty-five students (8 men) of the University of Cologne (mean age 21.92, *SD* = 2.08) participated in the experiment either for monetary compensation or for course credit. Each session lasted approximately 45 min.

### APPARATUS AND STIMULI

Apparatus, stimuli and the 2^nd^ order SRTT sequence were the same as in Experiment 1. The only difference concerned the across-task contingency manipulation.

### PROCEDURE

The overall procedure was the same as in the former experiments. Again, both tones occurred overall equally frequently across the dual-task training blocks. Crucially, each of the four SRTT target locations (1–4) now appeared with one particular tone with a probability of 75% – independently of its ordinal position within the SRTT sequence.

## RESULTS AND DISCUSSION

As in Experiments 1 and 2, trials were excluded due to SRTT errors (1.7%) or RTs < 200 ms or > 1500 ms in the SRTT (1.0%). Again, some trials fulfilled multiple exclusion criteria, thus overall 2.4% of the trials were excluded. We will, again, first report the results of the dual-task training phase and, second, the results of the single-task test phase. The mean IRIs were, again, positive (154 ms) indicating that the participants had predominantly responded to the SRTT first (see also ***Figure A1*** in the Appendix).

### PERFORMANCE IN THE TRAINING BLOCKS

***Table 3*** displays the mean RTs in the SRTT and in the tone-discrimination the task for the frequent (75% probability) and the infrequent (25% probability) SRTT-tone combinations as a function of block. Again, the participants became generally faster across the six training blocks in both tasks. In addition, the participants responded faster when they received the frequent combinations than when the infrequent combinations were presented.

**Table 3:** Mean RTs and SDs in the SRTT and the tone-discrimination task for the frequent (75% probability) vs. the infrequent (25% probability) SRTT-tone combinations as a function of block in Experiment 3.


SRTT-TONE COMBINATIONS	SRTT				TONE-TASK			
	
	FREQUENT (75%)		INFREQUENT (25%)		FREQUENT (75%)		INFREQUENT (25%)	
			
	MEAN	SD	MEAN	SD	MEAN	SD	MEAN	SD

Block 1	648	172	660	175	771	133	804	163

Block 2	618	160	637	161	764	157	783	149

Block 3	595	153	602	158	751	153	774	147

Block 4	591	155	596	166	747	143	765	154

Block 5	575	156	583	162	731	134	763	141

Block 6	554	135	557	143	720	139	733	156

Regular Block 8	435	69						

Random Blocks 7/9	445	61						

Learning Effect	9	19						


Accordingly, the two 6 (block) × 2 (tone frequency: frequent vs. infrequent) repeated measures ANOVAs (one for each task) with RTs as dependent variable revealed a significant main effect of block in the SRTT, *F*(5,120) = 19.21, *p* < .001, \eta _p^2 = .445, and in the tone-task as well, *F*(5,120) = 3.38, *p* = .035, \eta _p^2 = .123. Additionally, the effect of tone frequency also yielded a significant effect in the SRTT (9 ms), *F*(1,24) = 9.99, *p* = .004, \eta _p^2 = .294, and in the tone-task (23 ms), *F*(1,24) = 37.21, *p* < .001, \eta _p^2 = .608. Like in Experiment 2, the effect of the tone-frequency was additive to the block effect in both tasks (*F*s < 1 for the respective two-way interactions).

The SRTT error rates were quite low (1.28% and 1.50% together with the frequent vs. the infrequent tones, respectively) and did not differ across the blocks (all *F*s < 1).

### PERFORMANCE IN THE TEST BLOCKS

***Figure 2*** shows the results of the SRTT single-task test. As can be seen, the mean RTs of the collapsed random blocks 7 (2^nd^ half) and 9 were longer (9 ms) than those of the regular block 8. The respective (two-tailed) *t*-test revealed that this learning effect was significant, *t*(24) = 2.37, *p* = .026, *d* = 0.474.

This was also confirmed by the additional Bayes test (see Dienes, 2014). The Bayes factor was *BF* = 4.61 indicating that the participants had acquired knowledge about the sequence.

***Figure 2*** also shows that the effect in the SRTT error rates mirrored the RT effect. More errors occurred in the collapsed random test blocks than in the regular block (2.47% vs. 1.92%, respectively). However, the corresponding (two-tailed) *t*-test just failed the level of significance, *t*(24) = 1.79, *p* = .087, *d* = 0.357.

The findings of Experiment 3 reveal once again that during training, from early on, the participants responded faster in both tasks when the frequent SRTT-tone combinations were presented than when the infrequent pairings appeared. This suggests that the frequent pairings were expected. More importantly, we found preserved implicit sequence learning in the single-task test phase. This rules out that the 75% probability of the SRTT-tone pairings had generally hampered implicit sequence leaning. Rather, it hints at the relevance of learning the across-task contingencies first, before associative chaining across the trials is possible. If the implicit learning mechanism operated concurrently within- and across-trials, as Schmidtke and Heuer (1997) seem to suggest, we should have found substantial implicit learning effects in both Experiments 2 and 3 since the respective integrated sequences were identical in length.

Overall, the response times were quite long in Experiment 3, in the dual-task training phase as well as in the single task test phase. At the time being, we have no explanation for this finding. However, it does not seem to have altered the results of Experiment 3 with regard to the main focus on implicit sequence learning.

## GENERAL DISCUSSION

The objective of the present study was to better understand the role of across-task integration – or predictability – in dual-tasking. A common finding is that implicit sequence learning is disturbed when participants are asked to conduct the SRTT while concurrently having to respond to a random secondary task. Several different explanations have been proposed in order to account for this finding. On the one hand, the impairment might stem from parallel response selection (Schumacher & Schwarb, 2009). On the other hand, a prominent explanation is the task integration assumption of Schmidtke and Heuer (1997). They suggested that participants tend to integrate the events belonging to the two different tasks into one single sequence. If exclusively the SRTT follows an inherent regularity and the other task does not – or does, but the sequences are uncorrelated – the resulting integrated sequence becomes extraordinarily long. This, in turn, makes it highly unlikely that the participants are able to learn it within a temporally limited learning phase.

Based on a previous study of Röttger et al. (2019), we argued that one open question is how exactly such an integration mechanism might work. According to Schmidtke and Heuer (1997), it operates concurrently on the within- and the across-trial contingencies. In contrast, the findings of Röttger et al. suggested that participants primarily learn the contingencies between the SRTT-element and the tone (within the same trial), but not the contingencies between the tone and the next trials’ SRTT-element. The present study aimed at shedding some more light on this question.

In Experiment 1, we replicated the formerly found ordinal position learning. Each target location of the 8-element 2^nd^ order SRTT sequence (3-1-2-4-1-3-4-2) was once randomly and once fixedly paired with a certain tone. The single-task test revealed preserved implicit learning exclusively for the formerly fixedly, but not for the formerly randomly paired SRTT-elements – suggesting that the participants had learned associations between the ordinal sequence positions and the fixedly paired SRTT-elements. Thus, associative chaining of the SRTT-elements (i.e., across the trials) was entirely absent. That is, even though the SRTT-element itself, and also the fixed SRTT-tone pairing, predicted unambiguously the next trials’ SRTT-element, the integration mechanism did not seem to operate across trial boundaries. Otherwise, at least every SRTT-element following a fixedly paired SRTT-tone compound should have been learned. This, however, was definitely not the case for the randomly paired SRTT-elements.

The results of Experiments 2 and 3 support the primacy of learning the SRTT-tone contingencies. In both experiments, each particular SRTT-element was combined with a certain tone in 75% of the trials, and with the respective other in 25%, resulting in (probabilistic) SRTT-tone sequences of the same length in both experiments. The only difference between these sequences was that the SRTT-tone contingencies were realized either between the eight sequence positions and the tones (Experiment 2) or between the four target locations and the tones (Experiment 3). The results of the single-task test phase showed reduced implicit sequence learning effects when the sequence positions were bound to the tones. By contrast, when the target locations were bound to the tones, sequence learning was preserved. If, as is suggested by the task integration account of Schmidtke and Heuer (1997), learning took place concurrently within and across trials, we should have found approximately the same amount of implicit sequence learning in both experiments because both integrated sequences were of the same length.

Overall, the reported findings seem to support the assumption of Hazeltine and Schumacher (2016) that a major problem in dual-tasking might be that the participants do not develop separate task sets or “task files” for the two tasks. Without further instructions that might prevent the formation of a common task set for both tasks, participants seem to primarily learn the temporally most contiguous across-task contingencies. Nevertheless, even though this interpretation seems to be rather straightforward, the results are somehow at odds with two assumptions in the literature.

The first point concerns the ordinal position learning found in Experiment 1 (as well as in the Röttger et al., 2019 study). For instance, Schuck, Gaschler, Kreisler, et al. (2012) have shown that for ordinal position learning to occur, a salient anchor defining the ordinal positions of the SRTT sequence is necessary. The usual experiments in the research on ordinal position learning provide such an anchor signaling the beginning of a sequence loop. In addition, usually short sequences of only three to five elements are used. Since our sequence was longer and our manipulation did not provide any anchor, one possibility to reconcile our findings with these assumed constraints of ordinal position learning is to suspect that the fixed SRTT-tone pairs in the overall confusing sequence of fixedly and randomly paired SRTT-tone elements might have been particularly salient and as such might themselves have served as anchors (Baddeley, 1968; Houghton & Hartley, 1995).

The second point concerns the more important question why the participants at all learned the SRTT-tone pairings. The two tasks used in our experiments consisted of a visual-manual and an auditory-vocal task. Thus, neither the stimulus- nor the response modalities did overlap – an arrangement that is thought to maximize the content-dependent separation of the two tasks (see, e.g., Halvorson & Hazeltine, 2015; Liepelt, Strobach, Frensch, & Schubert, 2011; Schumacher et al., 2001). Moreover, in the field of implicit associative learning, evidence for cross-modal learning is controversial. On the one hand, the findings of many other researchers in this field suggest that implicit learning is modality specific and does not take place across modalities (e.g., Conway & Christiansen, 2006; Eberhardt, Esser, & Haider, 2017; Frost, Armstrong, Siegelman, & Christiansen, 2015; Haider, Esser, & Eberhardt, 2018; Kemény & Meier, 2016; Li, Zhao, Shi, Lu, & Conway, 2018; Walk & Conway, 2016). On the other hand, however, a few findings suggest that the formation of cross-modal associations is possible (e.g., Mitchel & Weiss, 2010; Seitz, Kim, van Wassenhove, & Shams, 2007; Taesler, Jablonowski, Fu, & Rose, 2019). For instance, Mitchel, Christiansen, and Weiss (2014) showed that the participants learned a sequence of integrated auditory and visual stimuli. However, they only did so when the two stimuli were interpreted according to the McGurk illusion, and thus were integrated into cross-modal compounds. Potentially, conceptualizing the stimuli as one compounded event (rather than learning an association between to stimuli perceived as independent stimulus events) is a necessary pre-requisite when two modalities are paired.

Yet, it is unclear whether the learning mechanisms underlying cross-modal integration differ from those underlying the learning of associations between stimuli from different modalities (Cunillera, Càmara, Laine, & Rodríguez-Fornells, 2010; Glicksohn & Cohen, 2013; Mitchel & Weiss, 2010, 2011). What is known, is that cross-modal integration highly depends on temporal factors (Romanski & Hwang, 2012; Talsma, Senkowski, Soto-Faraco, & Woldorff, 2010). If the temporal synchrony of the stimuli is violated, integration is hampered. By contrast, associations between related elements are learned even when they are separated by a longer time interval (Destrebecqz & Cleeremans, 2001). Thus, it is conceivable that the simultaneous presentation of the SRTT-elements and the tones had led to cross-modal integration of the stimuli in our experiments.

However, the assumption of such a cross-modal integration of the stimuli runs contrary to the findings of Freedberg et al. (2014). According to their results, temporal proximity between the stimuli and the responses alone is not sufficient in order to bias the participants to integrate stimuli across modalities. Additionally, the authors had to suggest the participants to conceptualize the tasks as belonging together before they could find evidence for cross-modal integration (see also Hazeltine & Schumacher, 2016). Since we did not explicitly suggest the participants to conceptualize the tasks as belonging together, it is unclear why they should have integrated the stimuli of the two tasks.

There are two subtle, but noteworthy, methodological differences between the study of Freedberg et al. and our present experiments which might offer an explanation for the deviating findings. In the Freedberg et al. study, the training phase contained single- and dual-task blocks. Furthermore, the response window of maximal 3000 ms elapsed as soon as both responses had been made. The next trial always started after an inter-trial interval of 500 ms. By contrast, in our experiments, we did not present any single-task blocks during the training. Furthermore, the trial duration was constantly set to 2000 ms. Thus, the participants might have always experienced a rather long pause of approximately 1200 ms between the second response in trial n and the next presentation of the two stimuli in trial n+1 (given that they needed approximately 800 ms to respond to both stimuli). This might have increased the salience of the simultaneous stimulus presentation leading to the learning of integrated SRTT- tone compounds.

A simple idea of how to conceptualize such an integration process is based on the prediction error (Rescorla & Wagner, 1972). In case of the fixed SRTT-tone pairs, the prediction error could decrease from trial to trial leading to the formation of cross-modal compounds. Also, when the SRTT target locations and the tones were paired with a probability of 75%, the prediction error could decrease across training. By contrast, when the contingencies between the SRTT-elements and the tones additionally depended on the respective sequence position, the prediction error could not decrease as each target location once predicted the high tone and once the low tone. Our results further suggest that only after having acquired such SRTT-tone compounds, associative chaining of the SRTT-elements could appear. In line with the findings of, for instance, Walk and Conway (2016), associative chaining might, thus, nevertheless work in a modality specific way. This means that the primacy of learning the SRTT-tone compounds does not necessarily imply that the participants had, then, associated chains of the entire compounds, for instance, in our Experiment 3. It is conceivable that they only learned the transitions between the SRTT-elements within the visual-manual modality.

In summary: At the time being, we conclude from our results that, at least when the two stimuli in a dual-task paradigm are presented simultaneously – and the instructions do not induce the separation of the task representations (see, e.g., Halvorson, Wagschal, et al., 2013) – the learning of transitions across-trials presupposes the acquisition of the within-trial compounds. Integration does not seem to take place concurrently within and across trials as Schmidtke and Heuer (1997) suggested – at least when the two stimuli are presented simultaneously.

### IMPLICATIONS FOR DUAL-TASKING IN GENERAL

The present three dual-task sequence learning experiments add to the existing research the finding that task integration or, more specifically, across-task contingency learning seems to operate, per default, on the most contiguous contingencies, namely those between across-task events within the same trial. If it is impossible to integrate the two tasks (e.g., due to the randomness of the second task), implicit learning is hampered.

It does not seem to be the parallel response selection, that disturbs implicit sequence learning, as is, for instance, suggested by Schumacher and Schwarb (2009). If this was the case, implicit learning should have been generally impaired in all three experiments. Rather, the predictability across the tasks seems to be the limiting factor (cf. Keele et al., 2003; Rah et al., 2000; Schmidtke & Heuer, 1997).

In the present study, we further specified this across-task predictability account of the often-reported detrimental effect of dual-tasking on sequence learning. The implicit learning paradigm used here, together with the manipulation of the across-task contingencies, allowed us to disentangle these two accounts. The findings suggest that the participants in a dual-task experiment might interpret the two tasks presented within one trial as belonging together. Concurrently, the fixed time window of 2000 ms per trial seems to signal a clear boundary (probably due to inhibition processes) thereby decreasing the strength of across-trial associations. However, further research is needed to test whether this is a general effect in dual-tasking.

In addition, our research shows that the amount of implicit sequence learning might serve as a marker of dual-tasking limitations that cannot be assessed when using two randomly sequenced tasks. Conceptually, this marker goes beyond established markers of dual-tasking limitations like, for instance, the PRP effect (e.g., Pashler, 1994) or the BCE (e.g., Hommel, 1998; Janczyk et al., 2014) because it rather directly reveals whether the two tasks are represented within one integrated or two separate task-sets.

## Data Availability

The software and the merged raw data are available at *https://osf.io/v3aqh/*.

## Footnotes

One group of participants in the study of Halvorson, Wagschal, et al. (2013), the “integrated group”, was told that they were doing one, multipart task, that is, playing the part of a hotel concierge. They were told that the auditory and visual stimuli resembled a simplified version of relaying messages to the hotel staff: The visual stimulus indicated the room that required service, and the auditory stimulus indicated the type of service. Thus, each pair of responses could be conceptualized as conveying a single request (e.g., housekeeping for Room 2). The other group, the “separate group”, was confronted with exactly the same stimuli but assigned them mentally to separate tasks.

Since in our dual-task paradigm a potential focus of the associative learning mechanism on across-task events implies that temporally close co-occurring events (within a trial) are focused (rather than temporally more separated within-task events, across trials), we want to note that there are possibly some interesting parallels to findings in the statistical learning literature. Here, for instance, Gómez (2002) suggested that so-called non-adjacent dependencies are hard to learn because associative learning might, per default, always operate on the temporally most contiguous (adjacent) events – and has to be directed to more separate contingencies by appropriate manipulations. However, there are important differences between our paradigm and the paradigms used in the statistical learning literature, for instance, the simultaneous stimulus presentation and the requirement to give two responses per trial. Thus, we recommend that it should be tested in future experiments whether these apparent parallels are sustainable. While there have been early demonstrations in single-tasking setups that it can be more difficult to acquire non-adjacent as compared to adjacent contingencies (cf. Curran, 1997), surprisingly remote contingencies (cf. Ebbinghaus, 1885/2013; Remillard, 2010) and even multiple contingencies (cf. Fiser & Aslin, 2002) can be acquired – at least in single-tasking setups.

In line with the reasoning that the impact of task integration on sequence learning in multitasking depends on whether the SRTT is paired with a task with predictable or unpredictable stimuli and responses, De Oliveira, Raab, Hegele, and Schorer (2017) documented that multitasking can also be beneficial for sequence learning in a motor tracking task – if tones in the secondary task could be used to predict upcoming turns in the tracking task.

When pairing a 6-element SRTT sequence with a 6-element tone-task sequence, one sequence loop spans 6 trials with 2 stimuli each resulting in a 12-element integrated sequence. When pairing a 6-element SRTT sequence with a 5-element tone-task sequence, one sequence loop spans 6 × 5 = 30 trials with 2 stimuli each resulting in a 60-element integrated sequence. Using a random tone-task sequence would, thus, result in an indefinitely long integrated sequence (cf. Schmidtke & Heuer, 1997).

The finding that sequence learning was similarly strong in all three conditions of Schmidtke and Heuer (1997), when assessed with single-task instead of dual-task tests, is in line with the assumption that not the acquisition – but only the expression of implicit sequence knowledge is disturbed by dual-tasking (Frensch, Lin, & Buchner, 1998; Frensch, Wenke, & Rünger, 1999). Additionally, Schmidtke and Heuer (1997) had used a SRTT sequence with some unique transitions between its elements meaning that one SRTT-element uniquely predicts its follower. It has been shown that sequences containing some unique (among otherwise ambiguous transitions, meaning that at least two successive sequence elements are needed to predict the next), i.e. so-called “hybrid” sequences can generally be learned under dual-tasking conditions (Cohen, Ivry, & Keele, 1990).

One sequence loop spans 16 trials à 2 stimuli, thus the integrated sequence has 32 elements.

Contrary to the sequence used by Schmidtke and Heuer (1997), the SRTT sequence used by Röttger et al. (2019) contained no unique transitions. Such 2^nd^ order ambiguous sequences have been shown to be hard to learn under dual-tasking conditions (Cohen et al., 1990). The finding of substantial sequence knowledge in the correlated tasks condition (Röttger et al., 2019; Experiment 2) hints strongly at an important role of task integration for implicit sequence learning in dual-tasking situations.

Eleven participants received a structurally identical SRTT sequence with a slightly different order of the four target locations (2F-4F-3R-1F-4R-2R-1R-3F).

This outcome seems, at first glance, to run contrary to findings that a spatial sequence can be learned in the presence of an additional uncorrelated (e.g., temporal) sequence (see Shin & Ivry, 2002). Such evidence, however, stems from single-tasking contexts. That is, in contrast to our dual-tasking experiments, the elements of the additional timing sequence did not require any response. This difference is of particular importance. A former study of Röttger et al. (2019; Experiment 3; listen only condition) supports this assumption by showing preserved sequence learning when the additional (random) auditory stimuli did not require a response.

## APPENDIX

### IRI DISTRIBUTION

In all three Experiments, the mean inter-response intervals (IRIs), computed as RT_tone-task_ – RT_SRTT_, were positive indicating that the participants had predominantly responded to the SRTT first. ***Figure A1*** shows the respective IRI distributions. As can be seen, IRIs smaller than 0 ms, indicating a reversed task order, occurred very rarely.

### FREQUENCY EFFECT IN BLOCK 1

In all three experiments, we found already in block 1 consistently faster RTs when, at a respective SRTT-position, the frequent tone appeared compared to when the infrequent tone appeared. Since we have no explanation at hand, we exploratorily followed up on this finding. Potentially, this finding reflects a quick adaptation to the pairings of the events in the two tasks (for a similar finding, see Zhao et al., 2020).

In our current setup, the frequent SRTT-tone pairings were, in the majority of the trials, a repetition from the last sequence loop (i.e. from trial n-8) or even from the last occurrence of the same target location (i.e. from trial n-3 or trial n-5; Experiment 3). Thus, it is conceivable that already the first few repetitions of particular SRTT-tone pairings strengthened the participants’ respective expectations, resulting in fast responses for re-occurring pairings.

Accordingly, we exploratorily analyzed the frequency effect in block 1 separately for trials in which the SRTT-tone pairings repeated vs. switched compared to their last occurrence. As the stimulus material was not construed for such an analysis, it was not possible to split block 1 into two halves in order to analyze the development of the frequency effects – because in this case several cells had missing values. To avoid too many missing values, we computed the repetitions vs. switches of the SRTT-tone pairings in each experiment on the same basis as the contingencies were manipulated. That is, in Experiment 2, we computed them on the basis of the 8 sequence positions (i.e., Lag 8) and in Experiment 3, we computed them on the basis of the 4 target positions (i.e. Lag3/Lag5, respectively). In Experiment 1, the fixed and random pairings were also manipulated on the basis of the 8 sequence positions. For the fixedly paired SRTT-elements, however, every pairing was to 100% a repetition, making it impossible to conduct the same analysis as in the other two experiments.

In Experiments 2 and 3, we analyzed the frequency effect separately for the two tasks and separately for repetitions and switches of the SRTT-tone pairings. This led to four separate *t*-tests each for Experiments 2 and 3 comparing RTs with frequent vs. infrequent SRTT-tone pairings in block 1 (i.e., analyzing the frequency effect) separately for trials in which these pairings repeated vs. switched according to their last occurrence (computed on the same basis as the across-task contingencies were manipulated, respectively). As can be seen in ***Table A1***, the frequency effects were consistently larger and more substantial in the repetition trials. Whether these repetition trials are indeed forcing the very early development of the frequency effects observed in the present experiments, should be investigated in future research.

**Table A1:** Mean frequency effects (i.e., RT with infrequent minus RT with frequent SRTT-tone pairings) in the SRTT and the tone-task in block 1 of the Experiments 2 and 3. These RT-differences were computed separately for trials in which the SRTT-tone paring repeated vs. switched according to the last occurrence 8 trials ago (Experiment 2; Lag8) or 3 or 5 trials ago (Experiment 3; Lag3/Lag5). The *p*-values stem from the respective two-tailed *t*-tests.

FREQUENCY EFFECT IN BLOCK 1	RTS IN THE SRTT						RTS IN THE TONE-TASK					
	
	EXPERIMENT 2			EXPERIMENT 3			EXPERIMENT 2			EXPERIMENT 3		
			
	(LAG8)			(LAG3/LAG5)			(LAG8)			(LAG3/LAG5)		
			
	MEAN	N	*P*	MEAN	N	*P*	MEAN	N	*P*	MEAN	N	*P*

SRTT-tone repetition	36	25	0.014	43	25	0.163	43	25	0.009	99	25	0.025

SRTT-tone switch	4	25	0.518	4	25	0.552	17	25	0.062	17	25	0.123
